# Mid-Holocene expansion of the Indian Ocean warm pool documented in coral Sr/Ca records from Kenya

**DOI:** 10.1038/s41598-023-28017-0

**Published:** 2023-01-14

**Authors:** Maike Leupold, Miriam Pfeiffer, Takaaki K. Watanabe, Nobuko Nakamura, Lars Reuning, Alina Blume, Tim McClanahan, Mchulla Mohammed, Herman Kiriama, Dieter Garbe-Schönberg, Andrea Schröder Ritzrau, Jens Zinke

**Affiliations:** 1 Geological Institute, RWTH Aachen University, 52062 Aachen, Germany; 2 Institute of Geosciences, Kiel University, 24118 Kiel, Germany; 3 KIKAI Institute for Coral Reef Sciences, Kikai Town, Kagoshima, 891‑6151 Japan; 4 Faculty of Science and Technology, Keio University, Tokyo, Japan; 5 Department of Earth and Planetary Science, University of Tokyo, Tokyo, Japan; 6 Wildlife Conservation Society, Mombasa, Kenya; 7 National Museum of Kenya, PO Box 82412-80100, Mombasa, Kenya; 8 Kisii University, PO Box 408-40500, Kisii, Kenya; 9 Department of Physics and Earth Sciences, Jacobs University Bremen, 28759 Bremen, Germany; 10 Institute of Environmental Physics, Heidelberg University, 69120 Heidelberg, Germany; 11 School of Geography, Geology and the Environment, University of Leicester, Leicester, LE1 7RH UK; 12 Molecular and Life Sciences, Curtin University, Perth, WA 6102 Australia; 13 Australian Institute of Marine Science, Townsville, QLD 4810 Australia; 14 School of Geography, Archaeology and Environmental Studies, University of Witwatersrand, Witwatersrand, South Africa

**Keywords:** Palaeoclimate, Environmental chemistry

## Abstract

Proxy reconstructions suggest that mid-Holocene East African temperatures were warmer than today between 8 and 5 ka BP, but climate models cannot replicate this warming. Precessional forcing caused a shift of maximum insolation from boreal spring to fall in the mid-Holocene, which may have favored intense warming at the start of the warm season. Here, we use three Porites corals from Kenya that represent time windows from 6.55 to 5.87 ka BP to reconstruct past sea surface temperature (SST) seasonality from coral Sr/Ca ratios in the western Indian Ocean during the mid-Holocene. Although the Indian monsoon was reportedly stronger in the mid-Holocene, which should have amplified the seasonal cycle of SST in the western Indian Ocean, the corals suggest reduced seasonality (mean 3.2 °C) compared to the modern record (mean 4.3 °C). Warming in austral spring is followed by a prolonged period of warm SSTs, suggesting that an upper limit of tropical SSTs under mid-Holocene conditions was reached at the start of the warm season, and SSTs then remained stable. Similar changes are seen at the Seychelles. Bootstrap estimates suggest a reduction in SST seasonality of 1.3 ± 0.22 °C at Kenya and 1.7 ± 0.32 °C at the Seychelles. SST seasonality at Kenya corresponds to present-day SST seasonality at 55° E–60° E, while SST seasonality at the Seychelles corresponds to present day SST seasonality at ~ 65° E. This implies a significant westward expansion of the Indian Ocean warm pool. Furthermore, the coral data suggests that SST seasonality deviates from seasonal changes in orbital insolation due to ocean–atmosphere interactions.

## Introduction

**Figure 1:**
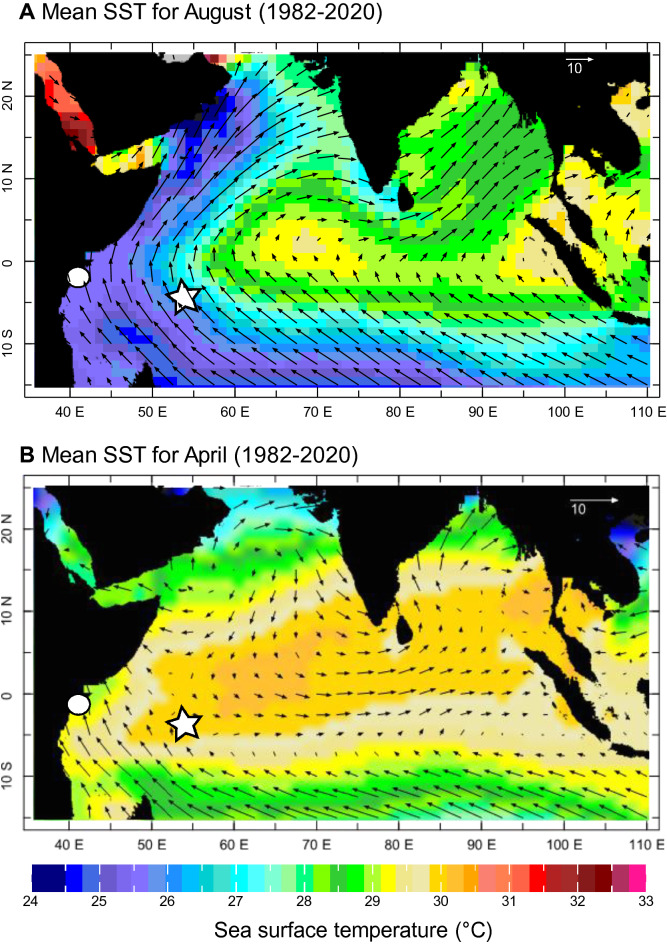
Monsoon circulation in the Indian Ocean. Mean SST in °C (colors) and surface winds (vectors) during (**a**) August and (**b**) April, averaged over 1982–2020. SST data is taken from AVHRR SST^25^, wind data from the NCEP/NCAR 40 reanalysis^67^. White dot: Kenya, star: Seychelles. Charts computed at IRI/LDEO Climate Data Library (http://iridl.ldeo.columbia.edu). Date accessed: 31 March 2021.

The Western Indian Ocean (WIO) is characterized by complex ocean–atmosphere interactions^1–3^, which are a potential source of non-linearity in the climate system that are at present poorly understood. The seasonal cycle of sea surface temperature (SST) in the WIO is impacted by the Asian monsoon circulation^1^. The SW monsoon develops in boreal summer (June–September), when the southern hemispheric trade winds cross the equator in the WIO, and form a continuous air stream extending into south Asia (Fig. 1), carrying large amounts of water vapor. Extensive precipitation occurs over India^1^. The SW monsoon leads to cooling along the path of the cross-equatorial air flow and to an abrupt drop of SSTs in the WIO^1^ (Fig. 1, Fig. S1).

**Figure 2:**
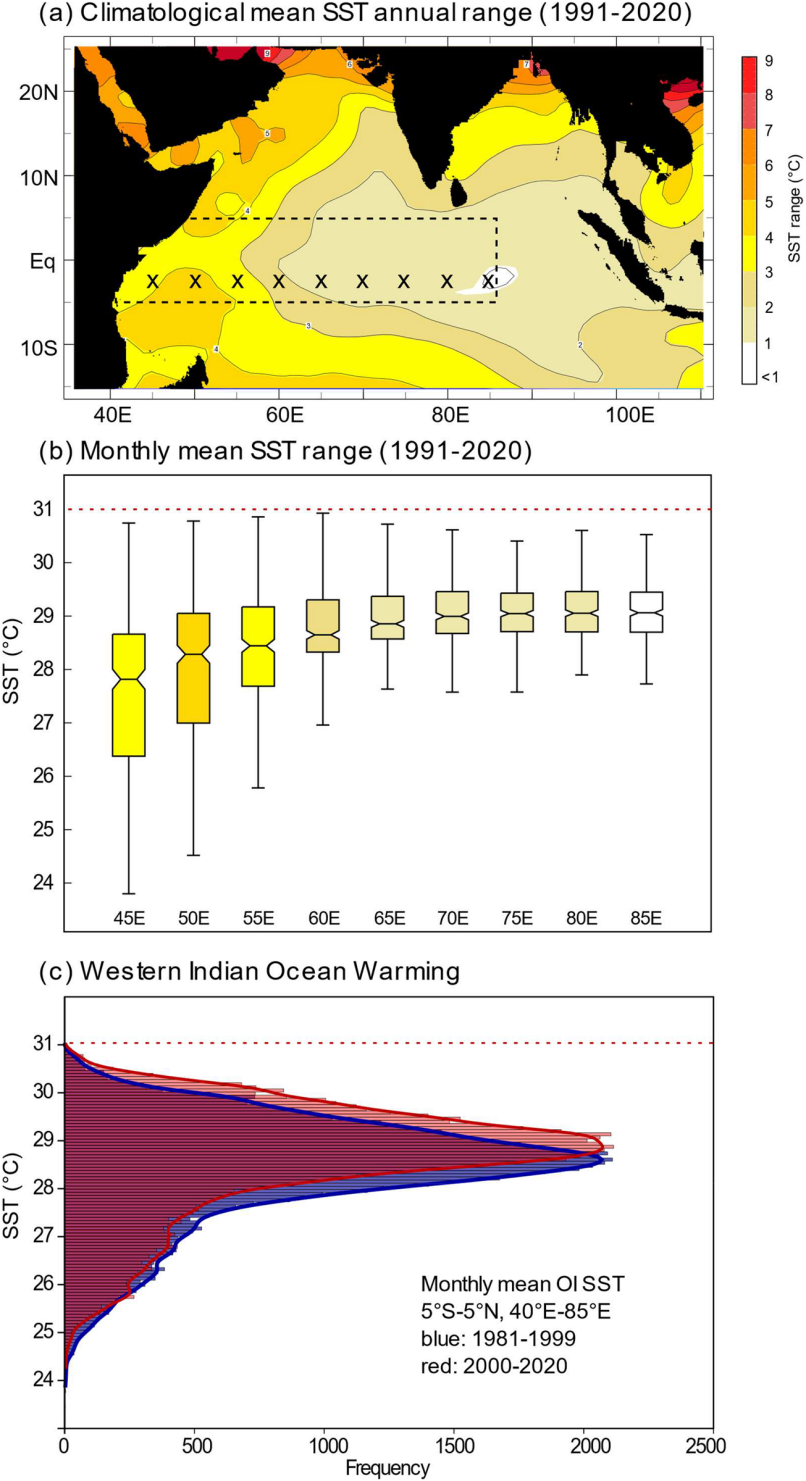
SST seasonality, range and warming in the WIO. (**a**) Map showing SST climatology in the Indian Ocean for the time period 1991–2020. SST data is taken from AVHRR SST^25^. Chart computed at IRI/LDEO Climate Data Library (http://iridl.ldeo.columbia.edu). Date accessed: 31 March 2021. (**b**) Boxplots showing range of monthly mean SSTs (AVHRR SST^25^, 1991–2020) in the WIO (grids where data was taken are marked with an ‘x’ in **a**). Boxplots show the median (horizontal line and notches), interquartile range and maximum/minimum values of monthly mean SST as whiskers. Maximum SSTs do not exceed 31 °C. Minimum SSTs are lowest in the path of the SW monsoon jet. (**c**) Histogram comparing the distribution of monthly mean AVHRR SSTs^25^ in the WIO (data was taken from area marked with dashed rectangle in **a**) for the time period 1981–1999 and 2000–2020. Note the shift towards warmer SSTs in the latter period and the rapid drop at 30–31 °C. AVHRR SST^25^ data was obtained from the IRI/LDEO Climate Data Library (http://iridl.ldeo.columbia.edu). Date accessed: 31 March 2021.

In boreal winter, the NE monsoon develops^1^. North-easterly winds from the cold Asian continent flow towards the warm, tropical Indian Ocean (Fig. 1). Maximum SSTs in the WIO occur in April/May^1^ and are very uniform (between 30 and 31 °C) from the East African coast to the eastern Indian Ocean warm pool (Fig. 2). Because of these uniform SST maxima in the WIO, the mean and median SSTs, as well as the seasonal ranges of SST are largely determined by SW monsoon cooling in boreal summer.

In response to the rise in anthropogenic greenhouse gases, the WIO is currently warming faster than any other ocean basin^4^. Warming occurs in all seasons, but warming rates are highest in the (cold) SW monsoon season^4,5^. As a result, the Indian Ocean warm pool expands^5–7^. A continuation of this trend is projected for the twenty-first century in response to anthropogenic warming^7^. However, although the warming of the WIO after 2000 is clearly seen in the distribution of monthly mean SSTs (Fig. 2), they do not rise above 31 °C. WIO SSTs show a skewed distribution with a sharp drop at 30–31 °C, and SST seasonality is lower in regions of high SSTs (Fig. 2). This skewed distribution of tropical SSTs originally led to the hypothesis of a dynamic thermostat mechanism that may limit SSTs in the tropical warm pool areas to a narrow range of ~ 2 °C (at present between 28 and 31 °C)^8^. While it is debated how maximum warm pool SSTs will change in times of rising atmospheric CO_2_ levels^7,9^, current warming patterns indicate that a warmer WIO will have a reduced range of SSTs, warmer minimum SSTs and reduced seasonality^10^.

The mid-Holocene (MH) is a key time period to investigate natural climate variability, as surface conditions and greenhouse gas concentrations were similar to preindustrial, while orbital configurations were different^11,12^. The latter changed the seasonal distribution of Earth’s insolation at the top-of-atmosphere^13^. Precession is the dominant orbital parameter in the tropics and modulates the seasonal cycle, causing a shift in the timing of maximum seasonal insolation^13^. The tropics are characterized by a semiannual cycle of insolation as the sun reaches its zenith twice a year. At present, insolation has two nearly equal maxima in March and September (Fig. S2). In the MH, the seasonal insolation maximum shifted to September and exceeded March insolation^13,14^ (Fig. S2). In the southern hemisphere tropics, maximum seasonal insolation therefore occurred at the start of the warm season. Holocene temperature reconstructions from the WIO and equatorial East Africa suggest a warm period in the MH with substantial, sustained, long-term warming between ~ 8 and 5 ka BP^15,16^, hereafter referred to as the MH WP.

Holocene coral Sr/Ca records provide time windows of past SST seasonality^14,17^. At sites with weak ocean–atmosphere coupling, several studies have shown a stable seasonal cycle of SST that closely tracks orbital insolation during the Holocene^14^. In contrast, Holocene SST seasonality in the WIO inferred from fossil Seychelles corals was very variable, indicating that non-linear ocean–atmosphere interactions modify the response to orbital forcing^17^. MH WP corals from the Seychelles suggested a pronounced reduction in SST seasonality.

Here, we present three new MH WP coral Sr/Ca records from the coast of Kenya, covering 11, 15 and 20 years. One new 15-year modern Sr/Ca record from Kenya was developed for comparison. The δ^18^O record of the modern coral was previously published and shown to record IOD variability^18,19^. In combination with the published coral data from the Seychelles, we will investigate zonal changes in MH WP SST seasonality in the WIO, and discuss implications for large-scale climatic changes in the tropical Indian Ocean.

## Regional setting and climate

### Present-day climate of Kenya

Kenya’s climate is influenced by the bi-annual migration of the Intertropical Convergence Zone. Seasonal rainfall maxima occur during the short rain period beginning in October and during the long rain period beginning in March^19,20^. The mean seasonal cycle of SST is very uniform along/off the Kenyan coast from 1° S to 3.5° S and 40° E to 45° E (Fig. S3). SST maxima occur in the two doldrums periods of December (28 °C) and March to April (29 °C) (Figs. S1, S3), with a shorter cooler period in between associated with a reversal of wind from the south to north^20^ and a shallowing of the thermocline (Fig. S4).

### Present-day seasonality and mean SSTs and in the WIO

In the WIO, maximum SSTs occur in boreal spring (March–May), following the spring insolation maximum, while the boreal fall insolation maximum translates into a smaller, secondary SST maximum in October-December (Fig. S5). In boreal summer (June–August), the SW monsoon induces pronounced, uniform cooling in the WIO. The magnitude of cooling decreases progressively towards the eastern Indian Ocean warm pool (Fig. 2, Fig. S6). SST minima are < 24 °C at 45° E, < 25 °C at 50° E and < 26 °C at 55° E (Fig. 2).

After warming in boreal fall, coastal upwelling off Kenya^2^, and open ocean upwelling at the Seychelles^3^ leads to a short (< 2 months) cooling period in January/February (Figs. S5, S6). Upwelling is driven by the development of the South Equatorial Counter Current in the boreal winter season, which causes coastal (Kenya) and open ocean (Seychelles) upwelling, the latter along a shallow thermocline ridge between 5° S and 10° S in the WIO^3^ (Fig. S4). In March–May, maximum SSTs reach 30–31 °C (Figs. 1, 2, Fig. S1).

Mean SSTs and SST seasonality in the WIO co-vary in the sense that warmer (colder) mean SSTs are associated with a reduced (increased) seasonality, because monsoon-induced cooling in July–August varies spatially and is strongest in the westernmost section of the tropical Indian Ocean, while maximum SSTs in boreal spring are uniform across the basin (Figs. 1, 2). At present, there is a strong linear relationship between mean SST and seasonality in the WIO (Fig. S7). Assuming that this relationship holds in the MH, a warming of the WIO should result in reduced SST seasonality. A sharp reduction in seasonality coupled with a strong increase in mean SSTs is seen between 55° E and 65° E (Figs. 1, 2, Fig. S6).

Interannual SST variability in the WIO is influenced by the El Niño Southern Oscillation (ENSO)^21^, which originates in the tropical Pacific, and the Indian Ocean Dipole (IOD), a coupled ocean–atmosphere interaction in the tropical Indian Ocean^21^. El Niño events lead to basin-wide warming in the Indian Ocean, which peaks in March–May with an average magnitude of < 0.5 °C^21,22^. Positive Indian Ocean Dipole events cause warming in boreal fall (September–November). The largest observed positive IOD event to date occurred in 2019 and led to a warming of < 1 °C in the WIO^23^. As a result, the mean seasonal cycle of SST is very stable.

## Results

### Modern and mid-Holocene coral Sr/Ca data from Kenya

The monthly coral Sr/Ca records of the Kenya corals are shown in Fig. S8. The modern coral KY16-1 covers the time period 1987–2002 and was calibrated with OI SST v2^24^ (1° × 1° grid) and AVHRR SST^25^ (0.25° × 0.25° grid) (Fig. S9, Table S1). Correlations are statistically significant for monthly mean and monthly anomaly records assuming six independent samples per year, which is a conservative estimate given our average sampling resolution of 10 samples/year (Table S1). The use of the high-resolution SST dataset does not influence the calibration results. The Sr/Ca-SST slopes are consistent with the mean and spread of the Sr/Ca-SST relationship established by^26,27^, justifying a temperature conversion using the mean slope (−0.06 ± 0.01 mmol/mol per 1 °C^27^). Centered SSTs inferred from monthly coral Sr/Ca data using the mean Sr/Ca-SST relationship show the same distribution as monthly satellite SSTs (Fig. S10).

**Figure 3:**
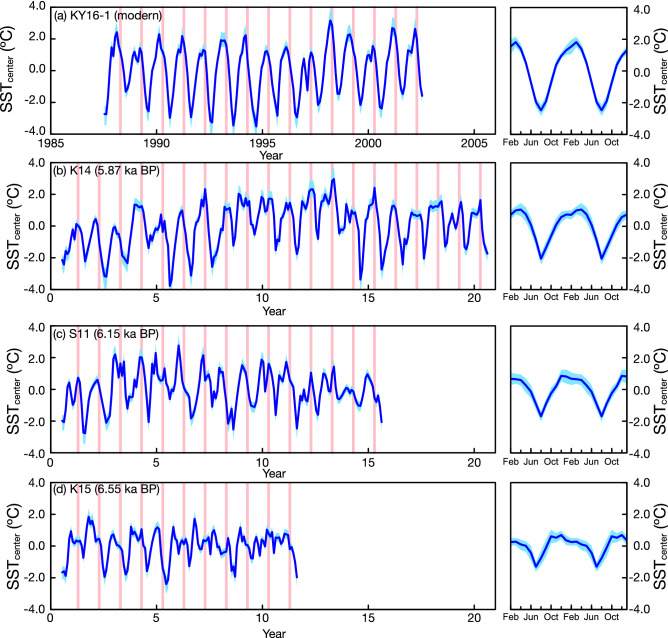
Modern and MH coral SST seasonality at Kenya. Monthly SSTs inferred from coral Sr/Ca (blue line, left) and mean seasonal cycles (right) calculated by averaging over all years for (**a**) modern coral KY16-1 and (**b–d**) the mid-Holocene corals S11 (**b**), K14 (**c**), K15 (**d**). Coral SSTs were derived from coral Sr/Ca by centering and conversion into SST units (°C) using a factor of −0.06 mmol/mol per 1 °C^26,27^. Uncertainty envelops (shading, 90% confidence intervals) for SST_center_ (left panels) were estimated using a Monte Carlo approach^27^ and included analytical and Sr/Ca-SST slope uncertainties . Uncertainty envelopes (shading, 90% confidence intervals) of the mean seasonal SST_center_ cycles (right panels) are estimated with a bootstrap method with 20,000 loops and include the distribution around the mean SST values of each month, the slope uncertainty of the Sr/Ca thermometer and the analytical uncertainty of coral Sr/Ca (the latter two contributions are small). Red shading marks April (boreal spring). Modern SST maxima (**a**) occur in boreal spring. The majority of SST maxima in the 6 ka BP corals occur in boreal fall (**b–d**).

Figure 3 compares monthly SSTs inferred from the Kenyan coral Sr/Ca records centered to their mean. Average seasonal cycles are estimated from the monthly data. In the modern coral record, SST maxima occur in boreal spring (March–April). The mean seasonal cycle is 4.3 °C (Fig. 3). The three MH WP corals comprise 11 to 20 years and were dated to 5.87 ka BP (K14), 6.15 ka BP (S11) and 6.55 ka BP (K15). They all show greater interannual variability in the timing of seasonal SST maxima, which occur in boreal fall (September–October) or spring (March–April). The average seasonal cycles indicate maximum SSTs in boreal fall (S11, K15) or spring (K14). The MH WP warm season is prolonged and lasts from boreal fall to spring in the following year. The prolonged warm season is possibly interrupted by a short cooling episode in January/February (Fig. 3). However, as the latter is short-lived and close to the limits of the temporal resolution of our records, we do not consider it further. In each MH WP coral, the mean seasonal cycle of SST is reduced compared to the modern record (< 3.5 °C in all cores) (Fig. 3). The reduction of the average MH WP seasonal SST cycle lies outside of the range of natural climate variability as seen in historical SST data covering the past 150 years, and as simulated in model data (Fig. S11).

### Modern and mid-Holocene SST seasonality at Kenya and the Seychelles

**Figure 4:**
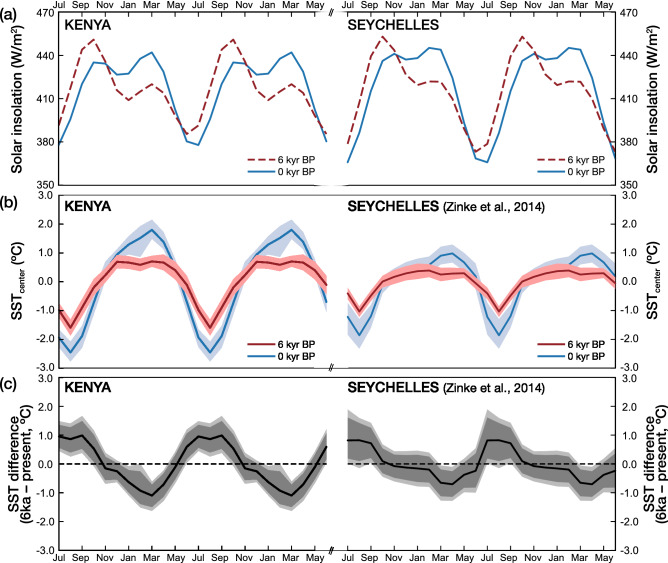
Insolation at the top of the atmosphere^28^ and mean seasonal SST cycles calculated from coral Sr/Ca. (**a**) Insolation at 6 ka BP (red dashed lines) and 0 ka BP (solid blue lines) for Kenya and Seychelles (**b**) mean annual SST cycles inferred from the mid-Holocene corals (red) and the modern corals (blue) from Kenya (left; this study) and the Seychelles (right, data from^17^. Colored shades are the 90% confidence intervals of mean SSTs in each month (see “Methods” for details). (**c**) Difference of mean seasonal SST cycles between the mid-Holocene and modern corals from Kenya (left; this study) and Seychelles (right, data from^17^). Dark and light grey shading indicates the 99 and 95% confidence level of the SST difference, respectively. Note that the SST difference was calculated from centered data, so negative (positive) SST differences do not indicate colder (warmer) absolute SSTs. Differences in mean seasonal cycles are significant when the confidence level of the SST difference is beyond the zero line.

Figure 4 compares the mean seasonal cycles of SST during the MH WP, as inferred from the coral Sr/Ca records from Kenya and the Seychelles, with seasonal insolation at the top-of-the-atmosphere^28^. While the overall range of the seasonal insolation remains similar, the insolation maximum shifts to boreal fall (October) in the MH, and exceeds the boreal spring maximum by 30 W/m^2^ at both sites.

**Figure 5:**
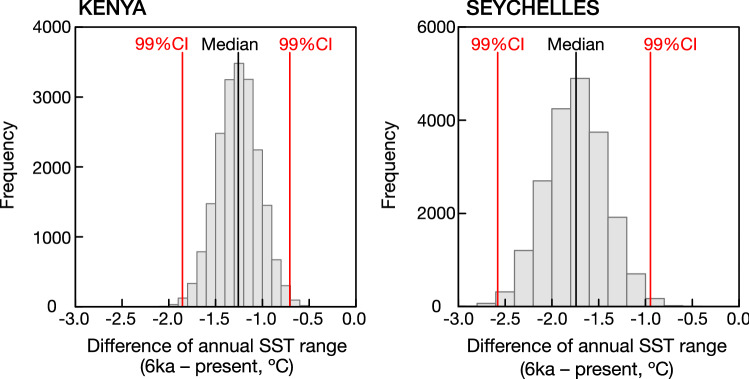
Reduction of SST seasonality in the mid-Holocene. Median difference (black line) with 99% confidence levels (red lines) between the mid-Holocene and modern SST seasonality estimated form Kenya (left; this study) and Seychelles corals (right, data from^17^). At both sites, the reduction in SST seasonality is statistically significant. We used the bias-corrected and accelerated (BCa) bootstrap method in R^29^ to assess the significance of our results (see “Methods” and Table S2 for details).

The coral Sr/Ca records from both sites show significantly reduced average seasonal cycles of SSTs, and a prolonged warm season that lasts from November-March, rather than the expected boreal fall maximum. The significance of the observed reductions in seasonality is evaluated using the BCa bootstrap method in^29^ (Figs. 4, 5, Table S2; see “Methods” for details). At Kenya (the Seychelles), the mean seasonal cycle reduces by ~ 1.3 ± 0.22 °C (~ 1.7 ± 0.32 °C) (Fig. 5, Table S2).

**Figure 6:**
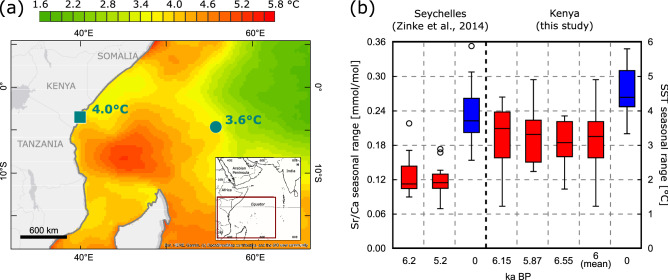
SST seasonality on the WIO, present-day and MH. (**a**) Map showing amplitudes of mean seasonal SST cycles in the WIO (1987–2002; AVHRR SST^25^). Charts computed using OpenStreetMap, date accessed: 31 March 2021. SST amplitudes average 4.0 °C (Kenya: blue square) and 3.6 °C (Seychelles: blue dot). (**b**) Boxplots comparing present-day and mid-Holocene seasonal amplitudes of coral Sr/Ca from the Seychelles^17^ and Kenya (this study). Median seasonal amplitudes are shown by the black solid line and boxes include 60% of the data. Whiskers indicate maximum ranges, open circles outliers. Corresponding SST ranges are shown on the right axis calculated using -0.06 mmol/mol per 1 °C^26,27^.

Figure 6 compares boxplots of modern and MH WP SST seasonality inferred from the Kenya and Seychelles corals. The boxplots show the medians of the seasonal cycles. Compared to the mean, the median is less sensitive to outliers, i.e. years with unusually high or low seasonality. At Kenya, the median present-day seasonal range is 0.26 mmol/mol (Sr/Ca) or 4.3 °C, compared to 0.19 mmol/mol or 3.2 °C in the MH WP. At the Seychelles, median SST seasonality reduces from 3.7 to < 2 °C. Each Seychelles coral contains 1–2 positive outliers, i.e. years where the seasonal cycle exceeds the interquartile range by > 1.5 standard deviations. In the case of the modern Seychelles coral, the outlier occurs during the coupled El Niño/Indian Ocean Dipole event of 1997/98, which led to unusually warm SSTs in the WIO and an increase in the seasonal cycle^17^.

## Discussion

### Mid-Holocene SST seasonality inferred from corals

The corals from the WIO indicate a significant reduction of MH WP SST seasonality, although the seasonal range of orbital insolation at the top-of-the-atmosphere remains similar. MH WP SST seasonality at Kenya (Seychelles) corresponds to present-day SST seasonality at 55° E–60° E (~ 65° E). Assuming a linear response to changes in orbital insolation, we would expect maximum MH SSTs in the WIO in boreal fall, i.e. at the start of the warm season, and the fall SST maximum should exceed the spring maximum by ~ 50%. However, the mean seasonal SST cycle inferred from the Kenyan corals show a prolonged warm season (Figs. 3, 4). Similarly, a prolonged warm season has been found at the Seychelles, located further east in the WIO, in two corals from 5.2 and 6.2 ka BP^17^, i.e. from the MH WP (Fig. 4). Our results suggest that systematic changes of SST seasonality occurred in the WIO that can be mapped using MH corals (Fig. 6). Compared with other Holocene coral studies from the literature, we note that sites with weaker ocean–atmosphere interactions may show a very stable SST seasonality that mainly tracks changes in orbital insolation (e.g.^14^). Understanding these relationships should become a focus for Holocene coral studies.

### Mid-Holocene temperatures in the Western Indian Ocean and East Africa

East African lake sediment records indicate warmer mean MH WP temperatures in equatorial East Africa between 8 and 5 ka BP^15,16,30,31^. A temperature reconstruction derived from a marine sediment core taken off the coast of Tanzania also indicates warmer SSTs (0.79 ± 0.22 °C) in the time interval between 7.8 and 5.6 ka BP^32,33^. Further evidence for a MH WP in the tropical Indian Ocean comes from tempestite deposits off SE Africa in the SW Indian Ocean, which are attributed to an increased storminess between 7.0 and 4.8 ka BP^33^. The deposition of these tempestites was suggested to reflect unprecedented tropical cyclone impacts, that are not seen today, and that are attributed to warmer SSTs in the WIO^33^.

Absolute coral Sr/Ca ratios track mean SST, but are also influenced by vital effects and/or skeletal heterogeneities, so that mean Sr/Ca ratios show a large spread at any given temperature^34–36^. The prediction error of mean SSTs derived from a single coral Sr/Ca record based on the calibration of^35^ is ~ 2.3 °C (Fig. S12). As we only have ≤ 3 coral samples per site that all grew during slightly different time periods in the MH WP, the uncertainties of mean SST changes inferred from our coral Sr/Ca records would be more than twice as large as the MH WP warming estimated by^32^. So, although it is possible to infer mean SST changes from coral Sr/Ca ratios when a sufficiently large number of coral samples is available^37,38^, we cannot do this in this study due to our small sample size. However, assuming that the relationship between lower seasonality and warmer mean SSTs seen today in the WIO (Fig. S7) and in the tropical oceans in general (Fig. S13) holds in the MH, the reduced seasonality inferred from our coral records would imply warmer mean MH WP SSTs in the WIO. This is confirmed by the sediment records from the WIO and East African lakes that show substantial, sustained warming between 8 and 5 ka BP^15,16,30,32^. Reduced seasonality coupled with warmer mean SSTs would imply greater warming in the cold season, e.g. in July–September.

### Mechanism for the response of mid-Holocene WIO SSTs to insolation

At present, maximum tropical Indian Ocean SSTs are between 28.5 and 31 °C (Fig. 2) and occur in boreal spring, at the end of the warm NE monsoon season (Fig. 1, Figs. S1, S5, and S6). Maximum SSTs are close to the upper limit of tropical SSTs^8^ (under present conditions), and are remarkably uniform across the Indian Ocean basin (Fig. 2). Paired coral δ^18^O and Sr/Ca records from modern Chagos corals (central Indian Ocean) indicated that an SST threshold of 28.5 °C strengthens atmospheric deep convection in the tropical Indian Ocean^39,40^. This would suggest that Indian Ocean SST variability is influenced by a tropical thermostat mechanism^8,9,40,41^, although the SST threshold for deep atmospheric convection as well as the upper limit of tropical SSTs may increase in times of warmer mean tropical SSTs^7,9^.

During the period covered by instrumental SSTs (~ 1880 AD-present), the WIO experienced a steady warming trend^4,42^. Although warming occurred in all seasons, it was strongest in the cold season (i.e., July–August, following the onset of the Indian summer monsoon), and seasonal SST minima warmed much faster than SST maxima^4^, leading to a reduction in seasonality. In the era covered by satellite SSTs (1982 AD-present), stronger warming in the SW monsoon season resulted in a visible reduction of SST seasonality in the easternmost section of the WIO^43^. Furthermore, a westward expansion of the Indian Ocean warm pool was observed^6^. During the warm NE monsoon season, warming rates are also slightly higher in areas where mean SSTs are lower^5^, and maximum WIO SSTs at present still do not exceed 31 °C (Fig. 2). This suggests that a warmer Indian Ocean will have a more uniform SST distribution and a reduced SST seasonality. We therefore believe that the reduced seasonality observed in our coral Sr/Ca records can be attributed to warmer mean temperatures in the WIO and equatorial East Africa between 8 and 5 ka BP, as inferred from sediment records covering the MH^15,16,30,32^. Furthermore, we propose that rapid warming in boreal fall driven by the fall insolation maximum raised SSTs at the start of the warm season to the upper limit of Indian Ocean SSTs under MH climatic conditions. Once SSTs reached this level, further warming was limited by a thermostat mechanism^8,41^, resulting in a prolonged, warm summer season. This scenario would explain (I) the reduced seasonality, and (II) the observation that the boreal fall SST maximum is not much larger than the spring maximum in the MH WP corals (contrary to our expectations based on insolation forcing), i.e. it would explain the deviation of SST seasonality in the MH warm interval from orbital forcing. A prolonged MH WP warm season in the WIO with temperatures above or close to the threshold for atmospheric deep convection would also explain the unprecedented tropical cyclone impacts off SE Africa^33^. (Note that the current increase in cyclogenesis in the SW Indian Ocean was shown to result from the warming of the WIO south of the equator^44^). However, as marine sediment cores do not have the temporal resolution to capture recent anthropogenic warming, it is currently unclear how MH SSTs in the western Indian Ocean compare to present-day SSTs. Nevertheless, we believe that a westward expansion of the Indian Ocean warm pool coupled with higher mean temperatures, a reduction in seasonality and a prolonged warm season is a plausible scenario for the MH WP.

### Mid-Holocene changes in monsoon circulation

Previous studies showed that the monsoon circulation is strongly influenced by orbital forcing^11,12^. In the MH, changes in obliquity caused an increase in boreal summer insolation (June–August) in the northern mid- to high latitudes and a decrease in boreal winter insolation^11,13^. This led to Northern Hemisphere warming and strengthened the SW monsoon^11^. Climate models also suggest a prolonged SW monsoon season in the MH^45^. A stronger MH Asian monsoon is documented in various geological archives indicating greater humidity and increased rainfall (e.g.^46^) and stronger upwelling in the western Arabian Sea (e.g.^47,48^). Stronger upwelling between 10.6 and 4.8 ka BP has been inferred from foraminiferal abundances in marine sediment cores from Oman^48^. Nitrogen isotope studies of sediment cores indicate stronger ventilation of the oxygen minimum zone during the mid-Holocene (8.2–4.2 ka BP) due to intense SW monsoon upwelling^47^. Given the pronounced SW monsoon-induced cooling in the WIO (Fig. 1), we originally expected to find an increase in SST seasonality in our MH WP coral Sr/Ca records, contrary to our results. However, warmer mean SSTs normally coincide with reduced SST seasonality (Fig. 2, Fig. S13), and a warm WIO was inferred from sediment cores between 8 and 5 ka BP^15,32^.

We note that the current warming of the WIO also leads to a reduction in SST seasonality^4,43^. However, since ocean warming is a slow process, the heat in the WIO has to build up over several decades to make a substantial difference and reach warm pool SST values of 28.0 °C or more^10^. The sustained western Indian Ocean warming has led to a greater spatial extension of the Indian Ocean warm pool in recent decades^6,10^, with a visible reduction in SST seasonality only in the easternmost section of the WIO^43^. However, the Indian Ocean will likely continue to warm in the future^10^. At present, the WIO warms faster than the Indian subcontinent, and SW monsoon rainfall over India declines^10^. However, anthropogenic aerosol emissions over South Asia may absorb solar irradiation and may slow the current warming over the Indian subcontinent^49^, so Eurasia may warm at a faster rate eventually^50^.

As sediment cores indicate that the interval from 8 to 5 ka BP was a period of sustained warming in the WIO^15,32^, our coral data from ~ 6 to 5 ka BP may offer a snapshot of WIO SST seasonality in a warm mean climate in that region. Since the warming was prolonged, the ocean was probably adjusted to these changes in boundary conditions. Our results thus suggest that a warm WIO (indicated by sediment cores) with reduced seasonality (inferred from the corals), which together imply warmer July–September SSTs, may co-occur with a stronger SW monsoon and higher rainfall over India. A stronger SW monsoon during the mid-Holocene has been documented in previous studies^11,46–48^.

This would be in line with predictions for future warming, which suggest that Indian summer monsoon rainfall will strengthen in response to anthropogenic warming of Eurasia, which is projected to exceed Indian Ocean warming eventually (e.g.^50,51^). Although contested, some studies also suggest that Indian rainfall is increasing again in recent years (e.g. after 2002)^51,52^.

## Implications and conclusions

We show that coral Sr/Ca ratios provide reproducible reconstructions of SST seasonality in the western Indian Ocean during the MH WP. Furthermore, our results show that spatial variations in SST seasonality can be traced using coral Sr/Ca records from two sites. Expanding this work would help to better understand the response of the tropical climate system to insolation forcing. Despite the reportedly stronger Indian summer monsoon in response to orbital forcing in the MH, our coral data show a reduced seasonality in the MH WP, suggesting a prolonged warm season and a westward expansion of the Indian Ocean warm pool. Seasonal SSTs deviate from seasonal insolation forcing, underlining the strong ocean–atmosphere coupling in the WIO. However, as marine sediment cores do not capture recent anthropogenic warming, it is still unclear how MH SSTs in the western Indian Ocean compare to present-day SSTs.

## Methods

### Coral collection

The modern coral sample KY16-1 was obtained in October 2002 from the Malindi Marine Park (3.2° S and 40.1° E) in Kenya^18^. The fossil corals were collected in November 2010 from the ruins of Swahili settlements, where they had been used as building material, on Lamu and Siyu Island, Lamu Archipelago, Kenya (2.2° S; 41.0° E). The fossil coral sample S11 was sampled from a collapsed pillar and a Mihrab at Shanga Friday Mosques, samples K14 and K15 were sampled from the Ungwana old and new mosque, where they were taken from the slope or buried in ground in the inner circle of the mosque. The fossil samples were cross-sectioned into 0.8–0.9 cm thick slabs and x-rayed (Fig. S14).

### Diagenetic screening

Samples were collected after a preliminary diagenetic screening in the field using a hand-lens. In total 23 apparently well-preserved coral samples were selected for further investigation using a combination of X-ray diffraction (XRD) and light microscopy (Fig. S15). The powder-XRD diffractometer at Rheinisch-Westfaelische Technische Hochschule (RWTH) Aachen University was calibrated to detect and quantify very low calcite contents above ∼ 0.2% following the method of^42^. In addition, the 2-D-XRD system Bruker D8 ADVANCE GADDS was used for XRD point-measurements directly on the coral slab with a calcite detection limit of ∼ 0.2%^53^.

From each coral sample, one to four parallel slabs were cut using a rock saw. In total 32 powder XRD samples, 92 2D-XRD samples, and 31 thin-sections were used to evaluate the degree of diagenetic alteration of these slabs. Eleven coral samples were subsequently selected for U/Th dating based on their excellent to good preservation (< 1% calcite and no or rare diagenetic textures in thin-section^54^). The U/Th analyses of six of these samples yielded Middle Holocene ages. The three best-preserved coral samples (S11, K14, and K15), were selected for climate proxy analysis.

Sample S11 was analyzed using one thin-section, one powder XRD and three 2D-XRD spot measurements. Sample K14 was analyzed using one thin-section, two powder XRD samples, and six 2D-XRD spot measurements. Two thin-sections, two powder-XRD, and six 2D-XRD measurements were used to analyze two slabs of sample K15 (Fig. S14).

All samples consist of 100% aragonite and no secondary aragonite crystals were observed in thin-sections. Samples K15 and K14 show slight darkening along some centers of calcification, indicating minor dissolution (Fig. S15). Directly adjacent (< 4 mm) to bioerosion traces the centers of calcification are surrounded by a brown hue, interpreted as incipient dissolution. Similar features have been observed in sample S11, where bioerosion traces show a brownish halo (< 3 mm) in thin-section. During sampling for climate proxy analysis, these areas adjacent to bioerosion traces have been avoided.

Overall, the samples show good to excellent preservation according to the criteria defined in^54,55^. Two representative microphotographs for each sample of the thin-section analysis are shown in Fig. S15.

### Subsampling and Sr/Ca measurements

Powder samples were collected from the modern coral KY16-1 at around 1 mm increments, which translates into a near-monthly temporal resolution^18^. From the fossil coral slabs, powder samples were collected at 1 mm increments for S11 using a precision drill grinder (type PROXXON FBS 12/EF) and at 0.5 mm increments for K14 and K15 using a micro-milling machine (type PROXXON FF 500 CNC). Table S3 compares the corals’ growth rates, the sampling resolution and the number of samples per year. The modern coral K16-1 has the lowest number of samples per year.

Sr/Ca ratio measurements were performed at Kiel University using a Spectro Ciros CCD SOP inductively coupled plasma optical emission spectrometer (ICP-OES). Elemental emission signals were simultaneously collected and subsequently processed following a combination of techniques described by^56,57^. Average analytical precision of Sr/Ca measurements as estimated from sample replicates was typically around 0.08% relative standard deviation (RSD) or less than 0.1 °C. All coral Sr/Ca ratios were normalized to an in-house standard. The international reference standard JCp-1^58^ was measured twice at the start and end of each OES run. JCp-1 had a median of 8.832 mmol/mol, and a standard deviation of 0.009 (1 sigma, N = 24) or 0.10% RSD^58^. Replication of coral Sr/Ca ratios was assessed based on corresponding samples along transect jumps (N = 14) of K15 and K14. The standard deviation of replicates is 0.03 mmol/mol. This includes analytical uncertainty, skeletal heterogeneity and sub-seasonal age uncertainties.

### Chronology development

The age model of KY16-1 was established based on the seasonal cycle of corals Sr/Ca, which shows distinct seasonal maxima (SST minima) between June and September. We assigned the highest Sr/Ca value to August 15 in each year, which is on average the coldest month. The actual timing of SST maxima at the sampling site (S 3°15.362'; E 40°7.983') as seen in NOAA OI SSTv2 data^24^ only deviates from August in three years, where it is shifted by one month (to July or September). We interpolated linearly between the age markers to get even-spaced time series with 12 data points per year. KY16-1 records 15 years, covering the period between 1987 and 2002.

The fossil coral samples S11, K14 and K15 were U/Th dated to an age of about 6.000 years. The uncertainties of the ages are approximately ± 64 years (S11), ± 68 years (K14) and ± 99 years (K15). For an overview on measured U/Th ages see Table S4 and S5. U/Th measurements were performed with a Finnigan MAT-262 RPQ Thermal Ionization Mass Spectrometry (TIMS) at the Institute of Environmental Physics, University of Heidelberg, Germany, following techniques described in^59^. Half-live values of Uranium and Thorium were taken from^60^. Following^61^, ages were corrected by assuming that corals contain a detrital component with Th/U ratio of 3.8 (average extraneous silicates) and with U isotopes in secular equilibrium and subsequently calculated the concentration of ^238^U and ^230^Th associated with the ^232^Th in the samples. Decay corrected ^234^U/^238^U activity ratios (δ^234^U_initial_) are calculated from the calculated ages. The δ^234^U_inital_ is used as U-Th age quality control. Ages are reliable having a δ^234^U_initial_ in the range of 146.8‰ (modern seawater^62^) ± 10‰ and are not reliable due to potential diagenetic overprint with values exceeding 146.8 ± 10‰. All three samples show closed-system behaviour and ages are reliable.

The sub-seasonal age models of the fossil coral samples were developed based on the seasonal cycles of coral Sr/Ca and by analyzing the density bands visible on x-ray images (Fig. S14). We assigned the highest Sr/Ca value to August 15. in each year, which lags the insolation minimum by 6 weeks, and interpolated linearly between these anchor points to obtain a time series with 12 equidistant time steps. The coral records cover 11 to 20 years.

Seasonal insolation curves were generated using the software PAST^63^ and the Asynchronous serial communication (ASC) insolation file of^28^. Monthly insolation means [in W/m^2^] were generated for the 6 ka BP (MH) period and for the present-day (0 ka BP) for Kenya’s Indian Ocean coastal strip (S 02°07.926; E 41°04.013) and the Seychelles following^17^.

### Coral Sr/Ca-temperature conversion and uncertainty estimates

The coral Sr/Ca records were centered, i.e. normalized with respect to their mean values^64^ and translated into SST units using the mean Sr/Ca-temperature dependence of -0.06 (± 0.01) mmol/mol per 1 °C established in^26,27^. This is consistent with the Sr/Ca calibration of the modern coral KY16-1 with satellite SST data (see Fig. S9). The uncertainties of SST_center_ inferred from Sr/Ca records were calculated using a Monte Carlo approach developed in^27^ and include the analytical uncertainty of Sr/Ca determinations and the slope uncertainty of the Sr/Ca-SST relationship (Fig. 3, left panels). We then estimated the mean values of SST_center_ in each month (= mean seasonal cycles) from the monthly Sr/Ca data. Uncertainty envelopes of the mean seasonal cycles shown in Fig. 3 (right panel) and Fig. 4 are estimated with a bootstrap method with 20,000 loops and included the distribution around the mean SST_center_ values of each month, the slope uncertainty of the Sr/Ca thermometer and the analytical uncertainty of coral Sr/Ca (the latter two contributions are small).

### Significance tests: (a) difference in MH and present-day seasonality

We estimated the (centered) mean SST in each month from the Sr/Ca records of the MH and modern corals from Kenya and Seychelles. We calculated the difference in centered SST between the MH and modern corals using a bias-corrected and accelerated (BCa) bootstrap method with the wBoot package and the software R version 4.1.2^29^. The significance test BCa bootstrap was applied to the difference in the seasonal SST range between the MH and modern corals. Results are significantly different when the errors do not overlap with 0 (Fig. 5).

### Significance tests: (b) MH SST seasonality vs. natural variability

We tested whether the observed difference in the seasonal SST range inferred from modern and MH coral Sr/Ca records from Kenya is beyond the range of noise resulting from natural-forcing using a Monte Carlo approach developed by^65^. Our hypothesis is that MH WP SST seasonality is different from present-day WIO SST seasonality as it arises from natural variability under present-day orbital forcing. We created SST records along the Kenyan coast using the Community Earth System Model (CESM) Last Millennium Ensemble (LME) Project (https://www.cesm.ucar.edu/projects/community-projects/LME/). We used ten simulated SST results from 850 to 1850 CE (in total, a 10,000 years-long record) and estimated the seasonal SST range from the LME SST record. A continuous 15-year record is randomly obtained from the LME-based seasonal SST range for the modern pseudo coral record. Three records with 11, 15, and 20 years long are randomly selected from the LME-based seasonal SST range to represent fossil pseudo coral records. We estimated the difference in the seasonal SST range between the fossil and modern pseudo coral records. We looped the estimate 30,000 times and described the histogram of this looped result. We then compared this histogram with the difference between the modern and MH coral SST seasonality (Fig. S11). We repeated this test using historical SST (ERSSTv5^66^) from Kenya (41° E, 3° S, 150 years of record). The result obtained using historical SST is comparable with the LME- simulation (Fig. S11).

## Supplementary Information

Supplementary Information.

## Data Availability

The coral datasets generated during the current study are available in the National Centers for Environmental Information Paleoclimatology repository (https://www.ncei.noaa.gov/products/paleoclimatology).
