# Supplementary material for: Mid-Holocene expansion of the Indian Ocean warm pool documented in coral Sr/Ca records from Kenya
Source: Sci Rep. 2023 Jan 14;13:777. doi: 10.1038/s41598-023-28017-0 (PMC9840608; doi:10.1038/s41598-023-28017-0)
Supplement: Supplementary file 1 — Supplementary Information. [file 41598_2023_28017_MOESM1_ESM.pdf]

Supplementary Information

1. Supplementary Figures

a. Climatological SST and surface wind field in the Indian Ocean

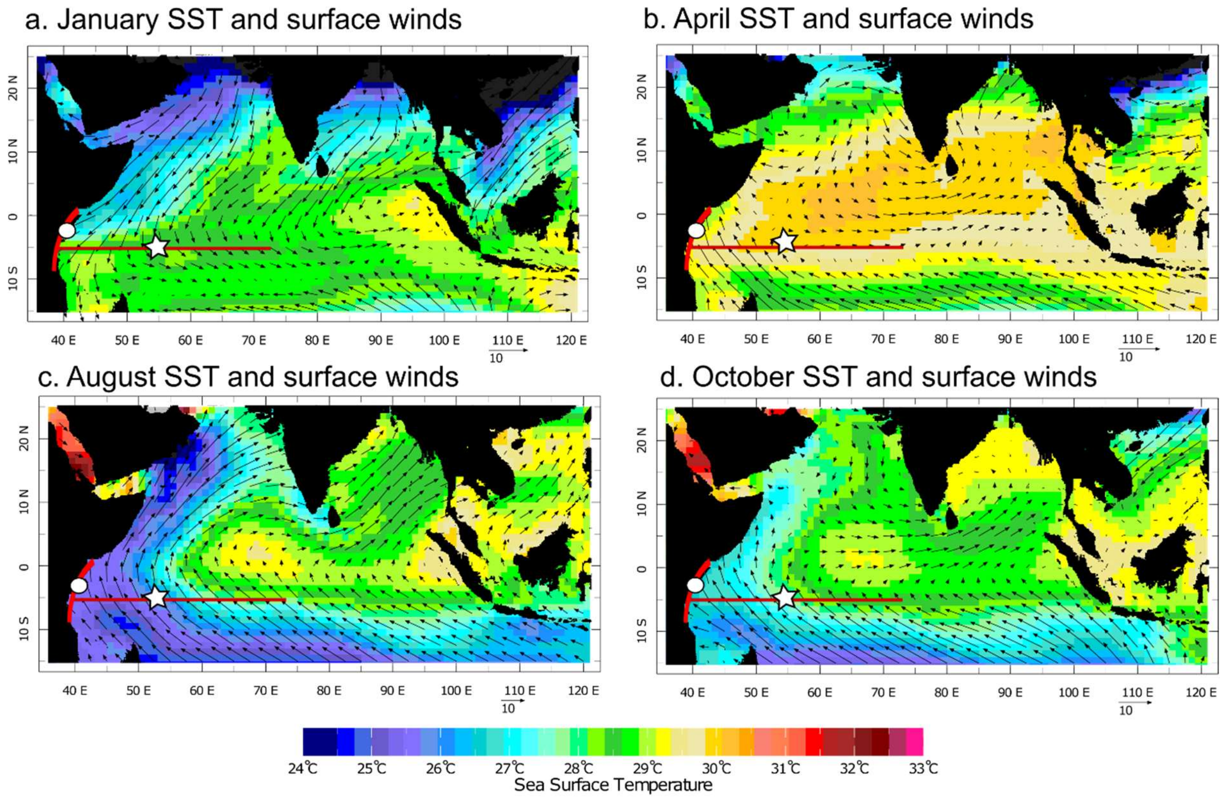

Figure S1: Climatological SST (colors, 1°x1° resolution, NOAA OISSTv2 [1] data) and surface winds [2] (vectors, 1000 mb, NCEP-NCAR CDAS-1) in the tropical Indian Ocean in January (a), April (b), August (c) and October (d). Charts computed at https://iridl.ldeo.columbia.edu/. Sample location of Kenya corals (circle) and Seychelles (star) are indicated. Curved red line marks section of climatological SST data shown in Fig. S5. Straight red line marks section of climatological SST data shown in Fig. S6.

b. Orbital changes in insolation from 6 ka BP to the present

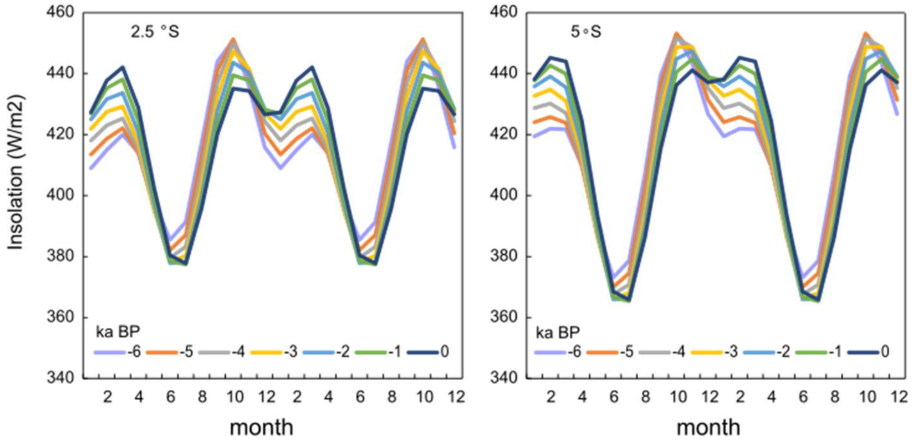

Figure S2: Mean seasonal cycle of orbital insolation at the top of the atmosphere [3] for 2.5°S and 5°S from 6 ka BP to the present. Note the shift in the seasonal insolation maximum from boreal spring (March-May, present) to boreal fall (October-December, 6 ka BP).

c. Mean seasonal SST cycles off the Kenyan coast

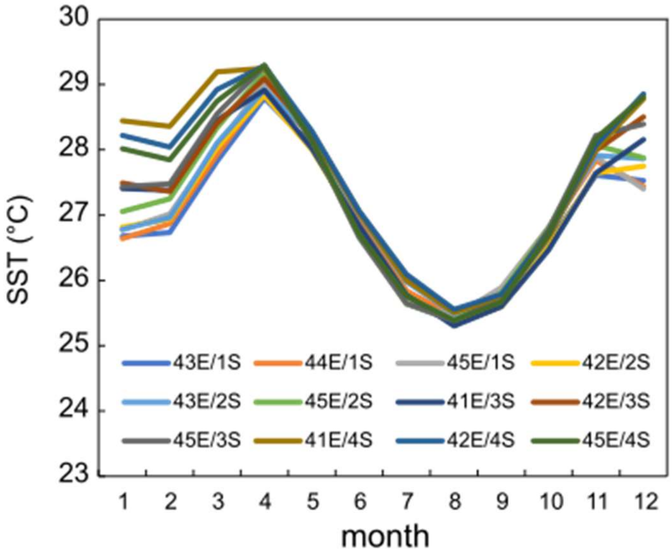

Figure S3: Mean seasonal SST cycles along the coast of Kenya as portrayed in satellite data (41°E-45°E, 1°S-4°S) Data is from the AVHRR SST product [4] (0.25 x 0.25° grid resolution), shown here at 1° steps.

d. Mixed layer depth, surface ocean currents and upwelling in the Indian Ocean

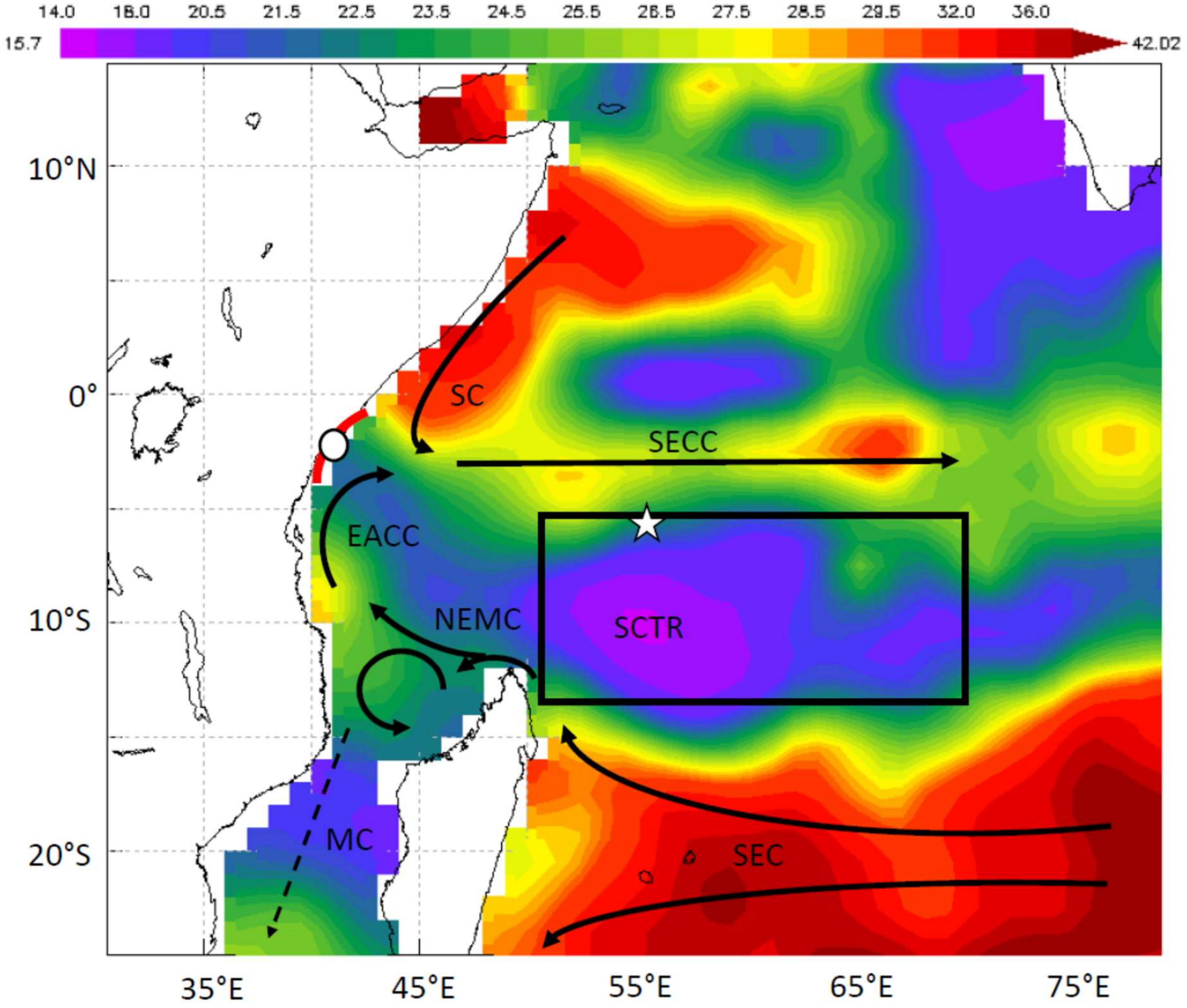

Figure S4: Mean mixed layer depth in March from ARGO float data (colors, chart computed at http://apdrc.soest.hawaii.edu/las86 on 28.0.6.2021). Northeast monsoon (boreal winter) surface ocean currents are indicated (adapted from [5]). SEC: South-Equatorial Current, MC: Mozambique current, NEMC: North-East Madagascar Current, EACC: East African Coastal Current, SECC: South-Equatorial Counter Current, SC: Somali Current. Black rectangle indicates zone of open ocean upwelling between 5°S and 10°S (Seychelles-Chagos thermocline ridge: SCTR; [6]). Thick red line marks upwelling region off the Kenyan coast [7]. Location of coral samples from Kenya (circle) and the Seychelles (star) are indicated.

e. SST seasonality along the East African Coast

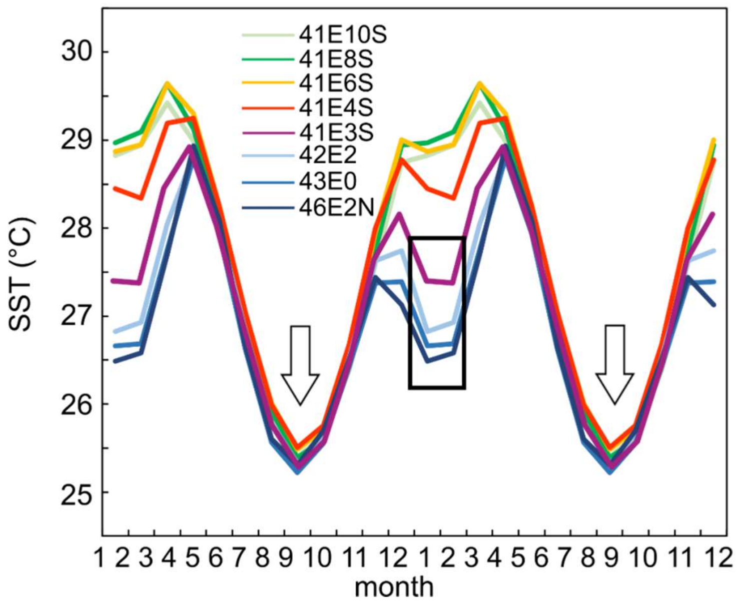

Figure S5: Climatological SST (NOAA OISSTv2 data [1]) along a N-S transect from 2°N to 10°S of the East African coast. Note the strong cooling following the boreal summer monsoon season (arrows, August-September) at all sites. Cooling during the Northeast monsoon coastal upwelling season (January-February) is seen between 3°S and 2°N and more variable (black rectangle). SST maxima occur in boreal spring and exceed 28.5°C at all sites (March-April).

f. SST seasonality in the south-western Indian Ocean (5°S, 41-75°E)

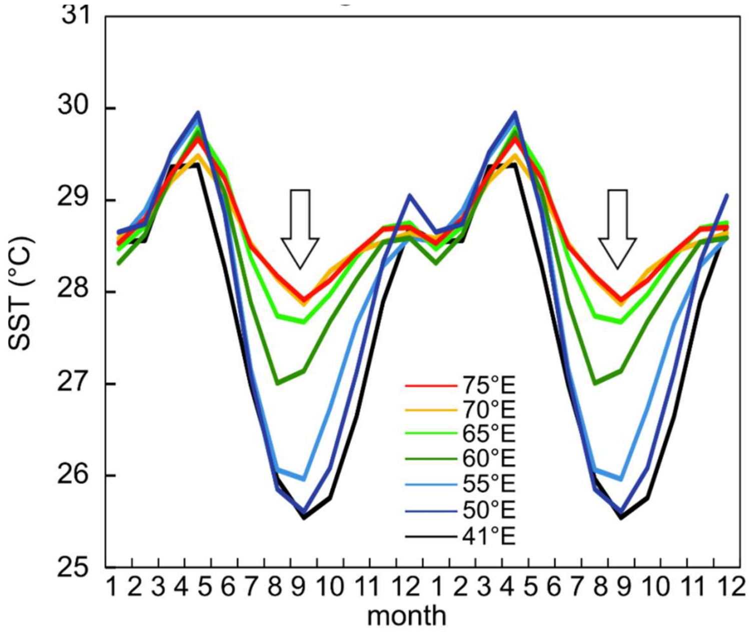

Figure S6: Seasonal cycle of SST in the Western Indian Ocean: climatological SST (NOAA OISSTv2 data [1]) along an E-W transect (41°E to 75°E) centered at 5°S. Note the strong cooling following the boreal summer monsoon season (arrow, August-September), which is strongest in the western Indian Ocean (41-55°E). All sites show a bimodal warm season with SST maxima in April (>29°C).

g. Seasonality and mean SSTs in the Western Indian Ocean

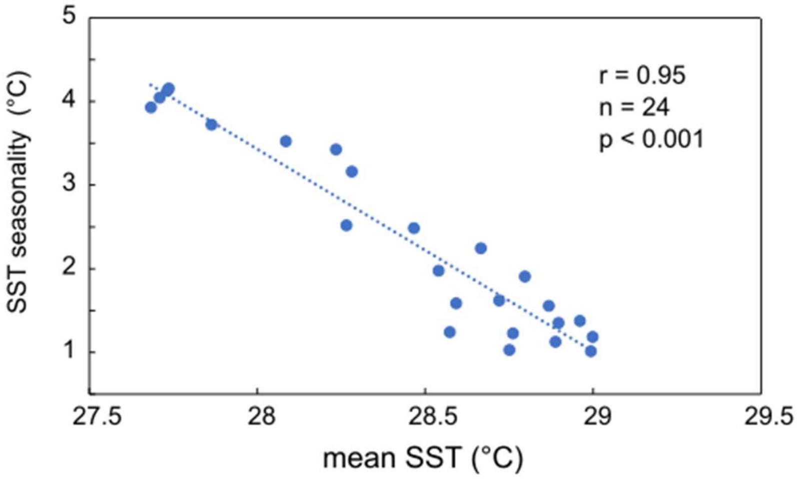

Figure S7: Ordinary Least Squares linear regression of mean SST and SST seasonality (climatological SST range) in the Western Indian Ocean (OI SSTv2 data, 1982-2020 [1]). Data is selected from the region 50°E-75°E, 2°S-5°S, using 5° (zonal) and 1° (meridional) steps.

h. Interpolated coral Sr/Ca data

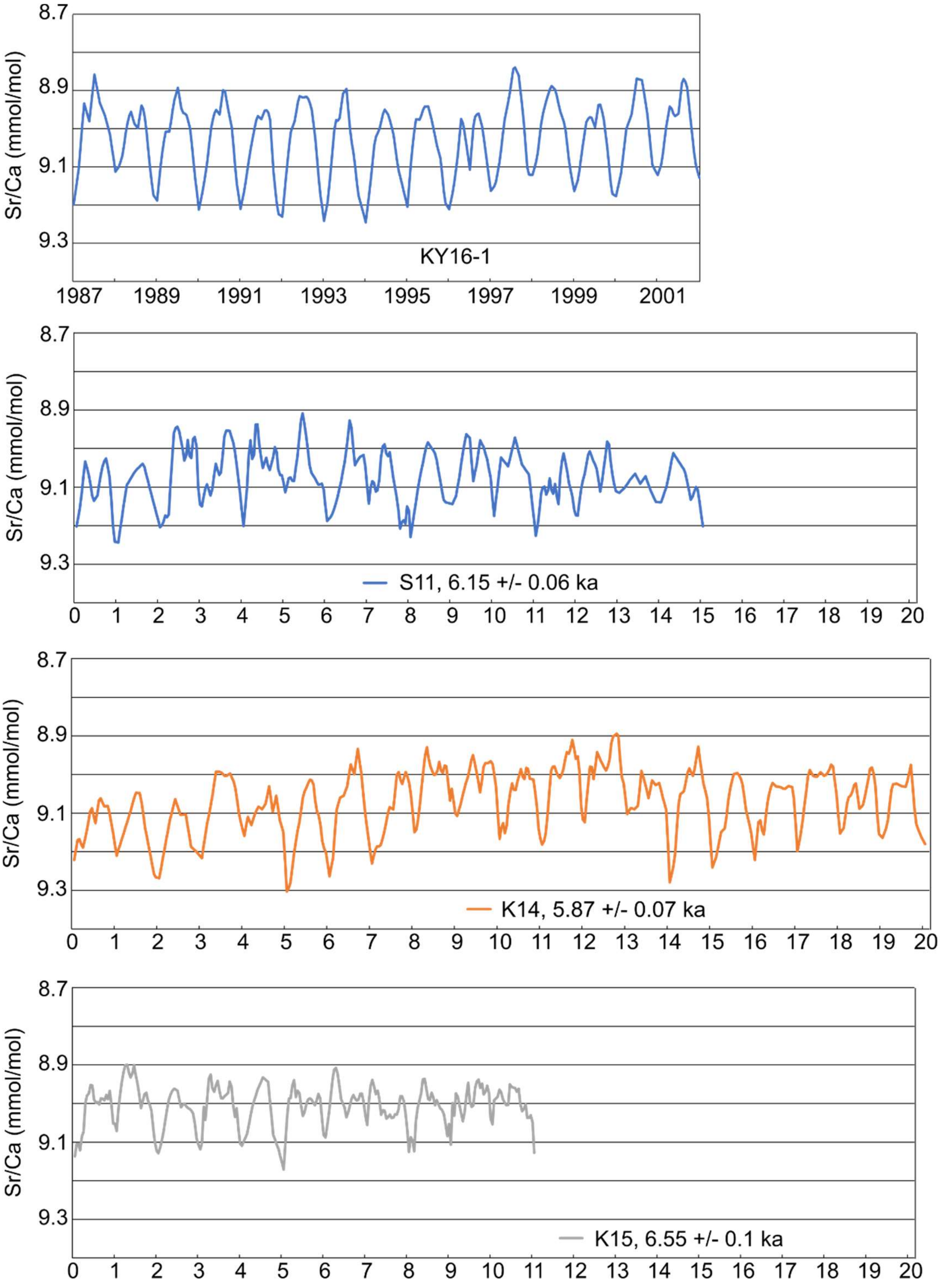

Figure S8: Monthly coral Sr/Ca ratios. K16-1: modern record, S11: 6.15ka BP, K14: 5.87 ka BP and K15: 6.55 ka BP.

i. Calibration of coral Sr/Ca with satellite SST

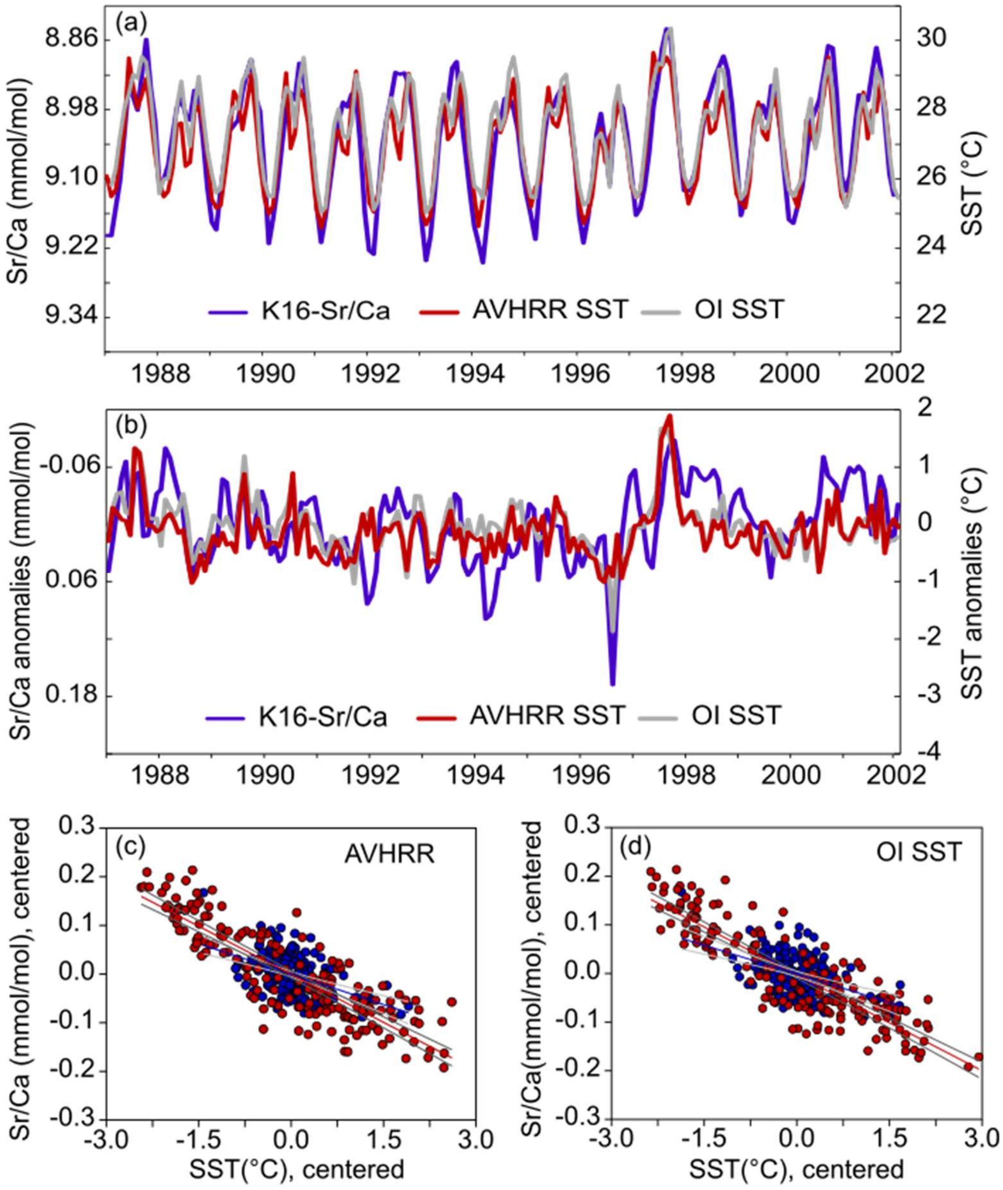

Figure S9: Correlation and regression of coral Sr/Ca of the modern KY16-1 sample with NOAA OISSTv2 [1] (1 x 1° grid) and AVHRR SST [4] (0.25 x 0.25° grid) data. (a) Plot of monthly coral Sr/Ca ratios (blue) with satellite SST (red and gray). All show distinct seasonal cycles. (b) Plot of monthly coral Sr/Ca anomalies (mean seasonal cycle removed) with satellite SST (red and gray). Note that coral Sr/Ca has been scaled so that -0.06 mmol/mol correspond to 1°C [8]. (c) Regression plot of coral Sr/Ca vs. AVHRR SST (red: monthly mean Sr/Ca vs. SST, time series centered to their mean, blue: monthly anomalies). The grey lines indicate the 95% confidence intervals of the regression lines. (d) Same as (c) but for OI SSTv22. Regression equations are listed in Table S1.

j. Distribution of monthly SSTs inferred from coral Sr/Ca and satellite data

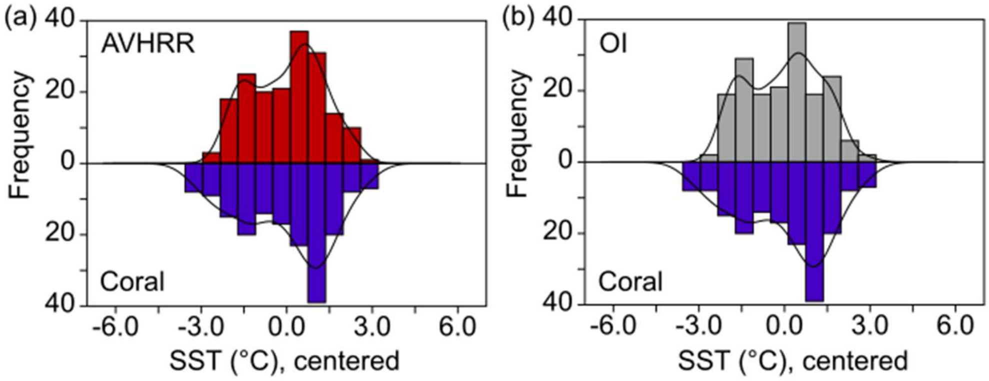

Figure S10: Distribution of monthly SSTs inferred from coral Sr/Ca (blue) compared with satellite SSTs (AVHRR [4], red and OISSTv2 [1], gray) for the time period 1987-2002. All time series were centered to their mean. Sr/Ca was converted to SST assuming a temperature dependence of -0.06 mmol/mol per 1°C [8,9].

k. Significance of MH reduction in seasonal amplitude at Kenya compared to natural climate variability of the past millennium

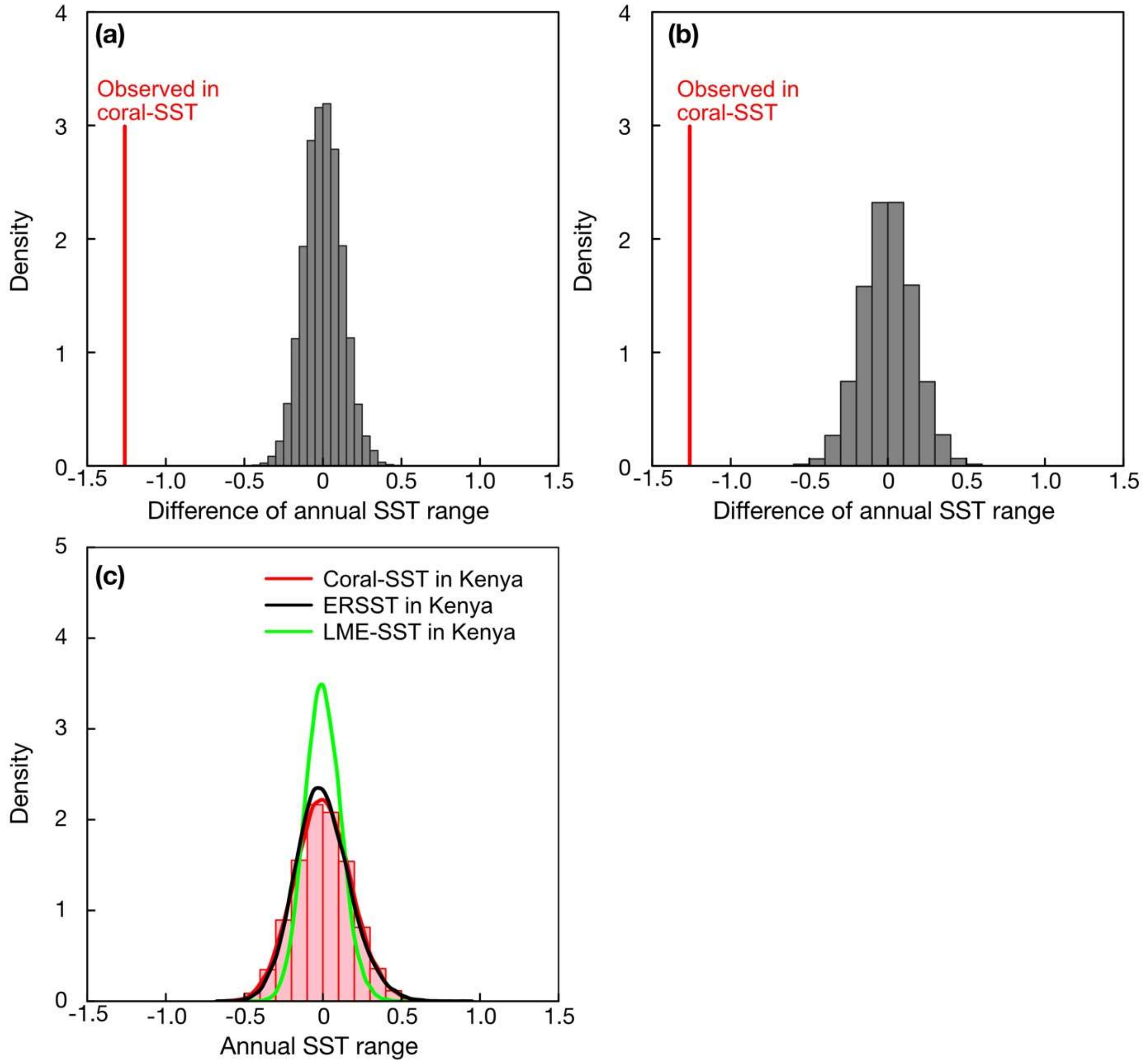

Figure S11: Significance test to assess the MH WP reduction in SST seasonality at Kenya against natural climate variability using a Monte Carlo method following [4]. (a) a histogram for the difference of annual SST range in the Kenya coast estimated from the pseudo dataset from LME simulation. The red line is the observed difference in the annual SST range calculated from the Kenyan coral Sr/Ca. (b) same as (a), using historical SST data (ERSSTv5, 41°E, 3°S, 150 years) from Kenya. (c) comparison of the seasonal SST range between modern coral-based SSTs (red), ERSSTv5 (black) and the pseudo dataset (green). Solid lines are the density curves. See Methods for details.

l. Mean coral Sr/Ca – SST regression and prediction error

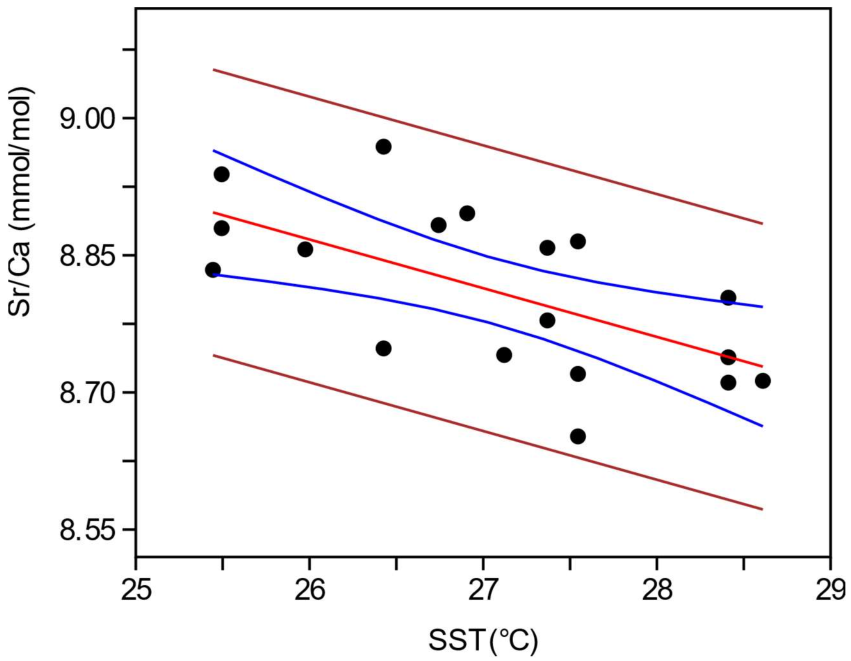

Figure S12: Ordinary least squares regression of mean Sr/Ca values versus mean satellite SST (data from Figure 1 of [10]). The regression equation is SST = −0.053(±0.017)*Sr/Ca + 10.25(±0.45); $r = -0.62$, $\sigma = 0.07$, $p < 0.01$, $n = 18$ (bright red line; blue lines are 95% confidence levels) (see [10] for details). Dark red lines: 95% forecast confidence levels for mean Sr/Ca ratios of single coral samples estimated in PAST [11]. The 95% CI levels for forecasts are $2*\sigma=2*0.07=0.14$ mmol/mol, which would correspond to an SST uncertainty of 2-2.9°C based on a Sr/Ca-SST relationship of -0.06±0.01 mmol/mol per 1°C [8].

m. SST seasonality in the tropical oceans

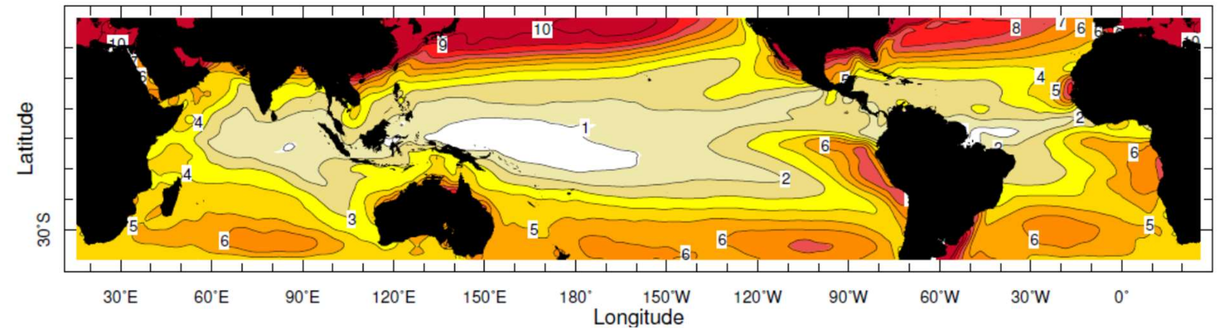

Figure S13: SST seasonality in the tropical oceans (NOAA OISSTv2 data [1]). Note the low seasonality in the warm pool regions. In the Indian Ocean, SST seasonality decreases from west to east. Chart computed at IRI/LDEO climate data library.

n. X-ray images of coral samples and sub-sampling paths

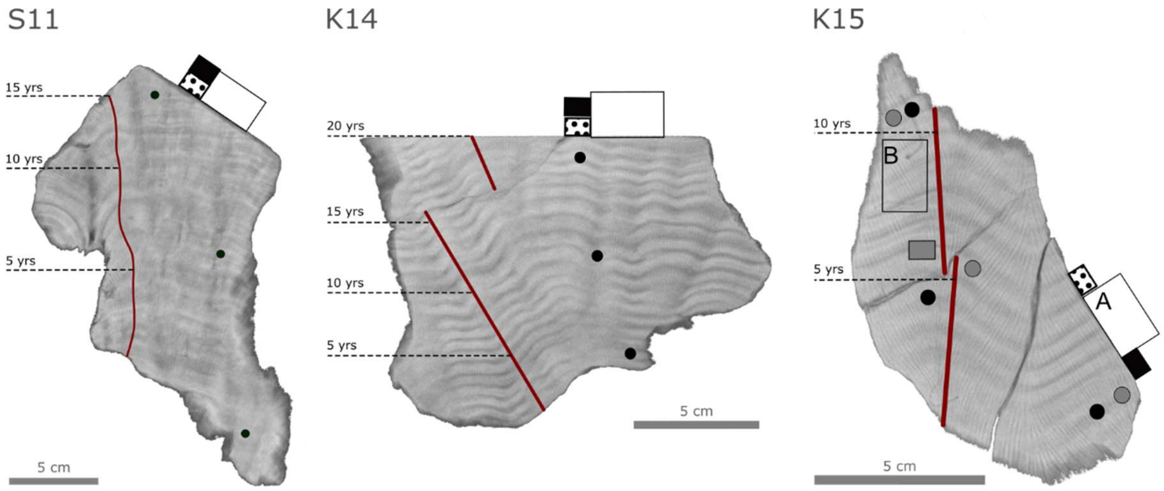

Figure S14: X-ray images of coral samples analyzed in this study. Age models were interpreted by counting the annual density bands combined with assigning each year's Sr/Ca maximum to the climatological SST minimum. Red lines indicate subsampling paths for proxy analysis. Black dots indicate position of 2D-XRD measurements. Grey dots on sample K15 indicate position of 2D-XRD measurements taken on a parallel slab of the coral, projected onto the radiograph. Black rectangles indicate position of conventional powder XRD measurements. Grey rectangles on sample K15 indicate position of powder-XRD measurements taken on a parallel slab of the coral, projected onto the radiograph. Large open rectangles indicate position of blocks that were used for thin-section preparation. Rectangle B was taken on a parallel slab of sample K15 and projected onto the radiograph. Stippled rectangles indicate position of samples for U/Th measurements.

o. Thin section images

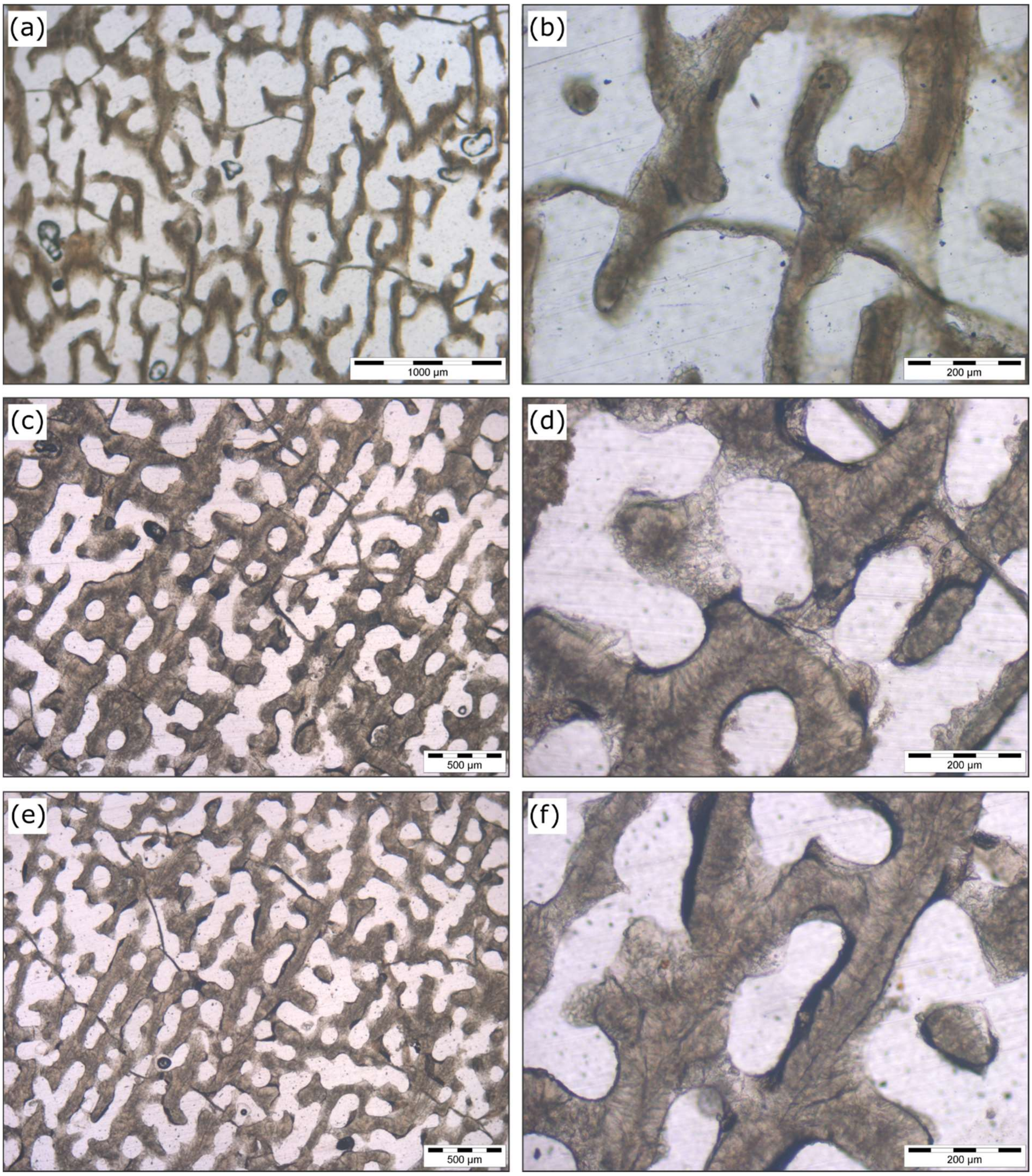

Figure S15: Representative thin-section photographs of coral samples S11 (a, b), K14 (c, d), and K15 (e, f). The corals show good to excellent preservation and contain neither calcite nor aragonite cements. Slight darkening along some centers of calcification indicates minor dissolution in all samples.

2. Supplementary Tables

a. Sr/Ca-SST regression

Table S1: Linear ordinary least squares regression equations and coefficient between near-monthly coral Sr/Ca, NOAA OISSTv2 [1] and AVHRR SST [4] for the grids including the sampling site of the modern coral (S 3°15.362'; E 40°7.983') for the time period 1987-2002. 95% confidence levels were calculated using bootstrap methods (N=1999). r is the correlation coefficient, p is the p-value, dof are degrees of freedom (based on a conservative estimate of 6 independent samples per year for the 15 year coral record). The mean equation from [9] is shown for comparison. All regression equations were computed with PAST [11].

core K16-1	Regression equation Sr/Ca= a x SST + b	95% CI a	95% CI b	r	p	dof
Monthly						
AVHRR	Sr/Ca = - 0.066 x SST +10.811	-0.071/-0.060	10.664/10.946	-0.86	<0.001	90
OI SST	Sr/Ca = - 0.066 x SST +10.826	-0.071/-0.060	10.699/10.949	-0.88	<0.001	90
Anomalies						
AVHRR	Sr/Ca = -0.042 x SST -0.0002	-0.054/-0.030	-0.005/0.005	-0.47	<0.001	90
OI SST	Sr/Ca = -0.039 x SST -0.0009	-0.052/-0.026	-0.006/0.004	-0.45	<0.001	90
Corrège, 2006	Sr/Ca = -0.0607 x SST +10.55					

b. Difference of mean seasonal SSTs cycles, present-day minus mid-Holocene

Table S2: Summary of bootstrap test results shown in Fig. 5 to estimate the difference between present-day and MH coral SST seasonality including 99% confidence levels (BCa bootstrap, R [12], see Methods section for details).

Bootstrap summary	Replications	Median	SE	99% CI
Kenya	20000	-1.26	0.22	(-1.86, -0.71)
Seychelles	20000	-1.74	0.32	(-2.58,-0.95)

c. Coral growth and sampling resolution

Table S3: Average annual growth rate, sampling resolution and number of samples per year of the modern and fossil corals from Kenya.

Sample	Growth rate (mm)	sampling resolution (mm)	samples/year
K16-1	9.6	1	9.6
S11	10	1	10
K14	5.7	0.5	11.3
K15	9	0.5	18

d. Th/U dating

Table S4 and S5: Overview on 230Th ages and corresponding years for fossil coral samples S11, K14 and K15. Dating was performed in 2011. Ages refer to measuring year. Uncertainties are 1 σ. See Methods for details.

Sample	^{238}U [µg/g]	Error [abso.]	^{232}Th [ng/g]	Error [abso.]	^{230}Th [pg/g]	Error [abso.]	Age (uncorrected)	Age (corrected)	
S11	3.0974	0.0031	2.817	0.017	3.184	0.032	6.175	6.152	± 0.064
K14	2.6758	0.0027	1.700	0.010	2.638	0.029	5.888	5.872	± 0.068
K15	2.5886	0.0026	7.377	0.095	2.858	0.037	6.622	6.551	± 0.099

LAB ID	Sample	^{238}U [µg/g]		^{232}Th [ng/g]		^{230}Th [pg/g]		Age [ka] (uncorrected)	Age[ka] (corrected)		$\delta U_{initial}$ [‰]	
IUP 5431	S11	3.0974	± 0.0031	2.817	± 0.017	3.184	± 0.032	6.175	6.152	± 0.064	143.9	± 1.8
IUP 5375	K14	2.6758	± 0.0027	1.700	± 0.010	2.638	± 0.029	5.888	5.872	± 0.068	148.9	± 2.4
IUP 5429	K15	2.5886	± 0.0026	7.377	± 0.095	2.858	± 0.037	6.622	6.551	± 0.099	148.1	± 2.0

References

1. Reynolds, R. W., Rayner, N. A., Smith, T. M., Stokes, D. C. & Wang, W. An Improved In Situ and Satellite SST Analysis for Climate. *J. Climate* **15,** 1609–1625 (2002).
2. Kalnay, E. *et al.* The NCEP/NCAR 40-Year Reanalysis Project. *Bull. Amer. Meteor. Soc.* **77,** 437–471 (1996).
3. Laskar, J. *et al.* A long-term numerical solution for the insolation quantities of the Earth. *A&A* **428,** 261–285 (2004).
4. Reynolds, R. W. *et al.* Daily High-Resolution-Blended Analyses for Sea Surface Temperature. *J. Climate* **20,** 5473–5496 (2007).
5. Schott, F. A., Xie, S.-P. & McCreary, J. P. Indian Ocean circulation and climate variability. *Rev. Geophys.* **47** (2009).
6. Vialard, J. *et al.* Cirene: Air—Sea Interactions in the Seychelles—Chagos Thermocline Ridge Region. *Bull. Amer. Meteor. Soc.* **90,** 45–62 (2009).
7. Jacobs, Z. L. *et al.* Shelf-Break Upwelling and Productivity Over the North Kenya Banks: The Importance of Large-Scale Ocean Dynamics. *J. Geophys. Res. Oceans* **125** (2020).
8. Watanabe, T. K. & Pfeiffer, M. A Simple Monte Carlo Approach to Estimate the Uncertainties of SST and δ 18 O sw Inferred From Coral Proxies. *Geochem. Geophys. Geosyst.* **23** (2022).
9. Corrège, T. Sea surface temperature and salinity reconstruction from coral geochemical tracers. *Palaeogeography, Palaeoclimatology, Palaeoecology* **232,** 408–428 (2006).
10. Cahyarini, S. Y. *et al.* Comment on "A snapshot of climate variability at Tahiti at 9.5 ka using a fossil coral from IODP Expedition 310" by Kristine L. DeLong, Terrence M. Quinn, Chuan-Chou Shen, and Ke Lin. *Geochem. Geophys. Geosyst.* **12,** n/a-n/a (2011).
11. Hammer, Ø., Harper, D.A.T., and P. D. Ryan. PAST: Paleontological Statistics Software Package for Education and Data Analysis. Available at https://palaeo-electronica.org/2001_1/past/past.pdf (2001).

12. R Core Team. R: A language and environment for statistical computing. Available at https://www.R-project.org/. (2021).
